# Using Genotyping by Sequencing to Map Two Novel Anthracnose Resistance Loci in *Sorghum bicolor*

**DOI:** 10.1534/g3.116.030510

**Published:** 2016-05-18

**Authors:** Terry J. Felderhoff, Lauren M. McIntyre, Ana Saballos, Wilfred Vermerris

**Affiliations:** * UF Genetics Institute, University of Florida, Gainesville, Florida 32611; † Genetics and Genomics Graduate Program, University of Florida, Gainesville, Florida 32611; ‡ Department of Molecular Genetics and Microbiology, University of Florida, Gainesville, Florida 32611; § Department of Microbiology and Cell Science—IFAS, University of Florida, Gainesville, Florida 32611

**Keywords:** anthracnose, *Colletotrichum sublineola*, GBS, sorghum

## Abstract

*Colletotrichum sublineola* is an aggressive fungal pathogen that causes anthracnose in sorghum [*Sorghum bicolor* (L.) Moench]. The obvious symptoms of anthracnose are leaf blight and stem rot. Sorghum, the fifth most widely grown cereal crop in the world, can be highly susceptible to the disease, most notably in hot and humid environments. In the southeastern United States the acreage of sorghum has been increasing steadily in recent years, spurred by growing interest in producing biofuels, bio-based products, and animal feed. Resistance to anthracnose is, therefore, of paramount importance for successful sorghum production in this region. To identify anthracnose resistance loci present in the highly resistant cultivar ‘Bk7’, a biparental mapping population of F_3:4_ and F_4:5_ sorghum lines was generated by crossing ‘Bk7’ with the susceptible inbred ‘Early Hegari-Sart’. Lines were phenotyped in three environments and in two different years following natural infection. The population was genotyped by sequencing. Following a stringent custom filtering protocol, totals of 5186 and 2759 informative SNP markers were identified in the two populations. Segregation data and association analysis identified resistance loci on chromosomes 7 and 9, with the resistance alleles derived from ‘Bk7’. Both loci contain multiple classes of defense-related genes based on sequence similarity and gene ontologies. Genetic analysis following an independent selection experiment of lines derived from a cross between ‘Bk7’ and sweet sorghum ‘Mer81-4’ narrowed the resistance locus on chromosome 9 substantially, validating this QTL. As observed in other species, sorghum appears to have regions of clustered resistance genes. Further characterization of these regions will facilitate the development of novel germplasm with resistance to anthracnose and other diseases.

Sorghum is the fifth largest cereal crop in the world by acreage grown, and is the third largest cereal crop grown in the United States (USDA 2014b; USDA 2014a). The United States was the largest producer of sorghum in the world during 2014, and was the single largest exporter of sorghum in 2013 (most recent available production data: FAO 2015). In 2012, sorghum production, imports, and exports represented a $1.4 billion industry within the United States (FAO 2015). The geographical area with the greatest increase in sorghum production in the United States is the Southeast, where sorghum acreage has risen nearly 75% over the past 10 yr (USDA 2004, 2014a). The hot and humid climate in the southeastern US provides an ideal environment for anthracnose infection (Crouch and Beirn 2009). Anthracnose is an aggressive disease that can be caused by a number of fungal species in different plant hosts. The causal pathogen of anthracnose in sorghum is *Colletotrichum sublineola* Henn. ex Sacc. & Trotter (Saccardo and Trotter 1913). Anthracnose can manifest itself in all aerial parts of the sorghum plant, either separately or in any combination of tissues (Pastor-Corrales and Frederiksen 1978). Anthracnose leaf infection is the most prevalent and most destructive (Figure 1), primarily through loss of total photosynthates produced by the plant, and is often the gateway stage for infections into other tissues (Harris *et al.* 1964; Ali and Warren 1987; Thomas *et al.* 1996). The infection of *C. sublineola* starts with adhesion and germination of the fungal conidia, which are dispersed via wind, surface water, or splash dispersal (Rajasab and Ramalingam 1989). Infection with this pathogen has been shown to cause up to a 70% yield reduction in susceptible sorghum lines, and the serious impact of this disease has been reported for over 60 yr (Luttrell 1950; Powell *et al.* 1977; Thomas *et al.* 1996). The pathogen is able to overwinter as mycelium, conidia, or acervuli in seeds, plant debris, or soil (Crouch and Beirn 2009), and can survive freezing temperatures. Genetic variation affecting virulence has been demonstrated to exist within *C. sublineola*, although a uniform classification system in which genotypes are grouped in distinct pathotypes does not (yet) exist (Ali and Warren 1987; Buiate *et al.* 2010; Prom *et al.* 2012). Pathotypes can vary between locations and years, depending on the selection of cultivar or hybrid, and crop management strategies (Prom *et al.* 2012).

**Figure 1:**
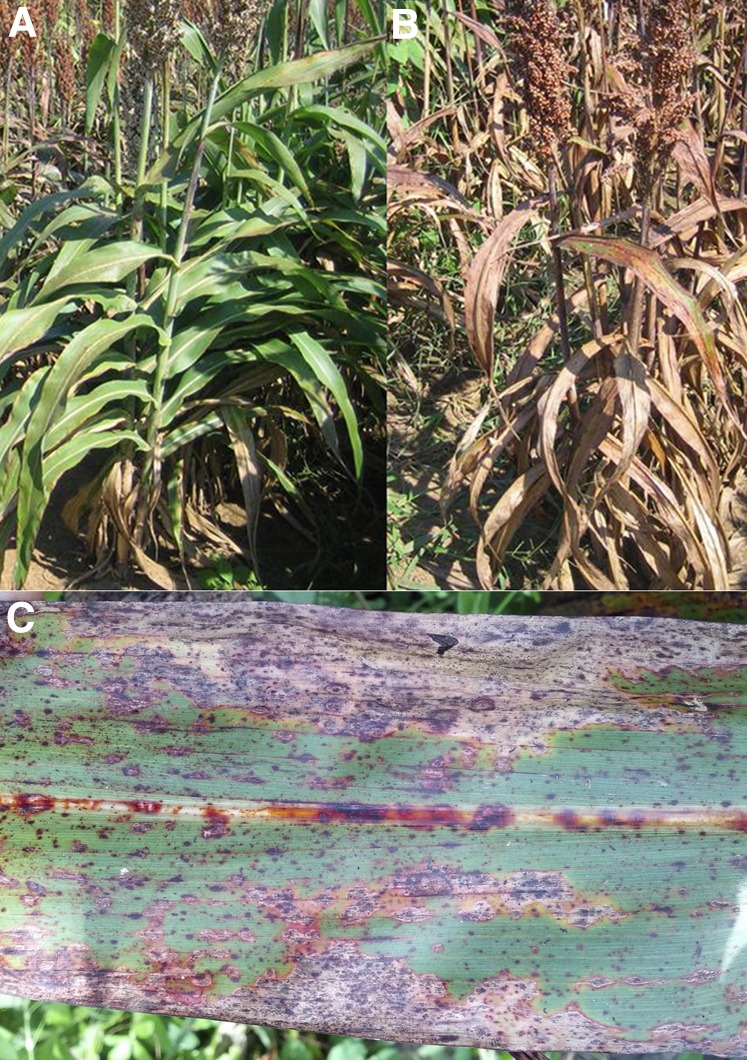
Disease phenotype under field conditions. (A) Cultivar ‘Bk7’ and (B) cultivar ‘Early Hegari-Sart’ in a University of Florida breeding nursery in Marianna, FL, where anthracnose was endemic. Both genotypes were of the same age. The brown appearance of Early Hegari-Sart is due to the presence of many lesions on stem and leaves. (C) Close-up of an Early Hegari-Sart leaf with anthracnose symptoms.

Genetic resistance is the most effective and cost-efficient measure to control anthracnose (Harris and Fisher 1973; Frederiksen 1984; Rezende *et al.* 2004). A plant’s ability to defend itself against pathogens relies on the recognition of conserved pathogen-associated or microbe-associated molecular patterns (PAMPs or MAMPs), or on the recognition of specific elicitors. Recognition of the former by nonspecific transmembrane pattern recognition receptors (PRRs) will set in motion a signal transduction cascade, *e.g.*, through the action of receptor-like kinases (RLKs), that ultimately leads to the defense against the pathogen. In contrast, recognition of specific elicitors via, for example, proteins containing a nucleotide binding site and leucine rich repeats (NBS-LRR), will lead to the direct transcriptional activation of defense-related genes mediated by the NBS (Dangl and Jones 2001; Martin *et al.* 2003; Jones and Dangl 2006; Hématy *et al.* 2009; Dodds and Rathjen 2010; Zipfel 2014). The genes that encode the latter class of proteins with both a receptor and effector domain are referred to as resistance (*R*-) genes, and are part of a larger group of defense-related genes that can also include genes encoding cell wall strengthening enzymes (Hématy *et al.* 2009; Sasidharan *et al.* 2011; Muthamilarasan and Prasad 2013; Bellincampi *et al.* 2015).

Different sources of anthracnose resistance have been identified in sorghum and mapped by several groups. Klein *et al.* (2001) mapped a quantitative trait locus (QTL) for anthracnose resistance on chromosome 6 using an F_5_ mapping population from a biparental cross between ‘Sureño’ and ‘RTx430’, evaluated in Texas using a natural infection. The QTL colocalized with QTL for resistance to grain mold or bacterial leaf stripe. Mohan *et al.* (2010) identified QTL for anthracnose resistance with a biparental mapping population of 168 F_7_ lines planted in two locations in India, using infected seeds as inoculum source in one location, and natural infection at the other. One QTL was identified on chromosome 4, and three on chromosome 6; the QTL on chromosome 6 overlapped with QTL for resistance against other diseases also observed during that study. This QTL colocalized with the resistance QTL reported by Klein *et al.* (2001), and contained the sorghum orthologs of known defense-related genes in maize and rice. Singh *et al.* (2006) identified a marker on chromosome 8 linked to a recessive anthracnose resistance allele, using a mapping population consisting of 49 F_8_ inbred lines that were selected based on extreme resistance or susceptibility to an anthracnose isolate from Hisar, India. Forty-six PCR-based random amplified polymorphic DNA (RAPD) markers were used for bulked segregant analysis (BSA), and a unique 1437 bp fragment was identified in the resistant parent and all resistant F_8_ progeny; this marker was mapped to the long arm of chromosome 8. Perumal *et al.* (2009) also used BSA to identify two markers linked to an anthracnose resistance locus on chromosome 5. Cuevas *et al.* (2014) generated a population of eight F_2_ families, and inoculated them with a *C. sublineola* isolate from Puerto Rico. An F_6_ recombinant inbred line (RIL) population of 50 lines was then created and inoculated with fungal isolates from Texas and Arkansas, leading to the identification of one marker on chromosome 5 linked to one or several closely linked resistance loci. Biruma *et al.* (2012) identified differentially expressed genes in two Ugandan sorghum genotypes (one resistant and one susceptible to *C. sublineola*) after anthracnose infection. Seven candidate genes were evaluated using virus-induced gene silencing (VIGS), leading to the identification of two *R*-genes encoding proteins with NBS-LRR domains, *Cs1A* and *Cs2A*, as the primary sources of resistance. Upadhyaya *et al.* (2013) used the sorghum minicore collection (Upadhyaya *et al.* 2009) to perform a population association study for anthracnose resistance. The 242 accessions were partially sequenced, aligned to the sorghum reference genome, and software was used to identify 14,739 single nucleotide polymorphism (SNP) markers. The association analysis identified eight loci linked to anthracnose resistance; four on chromosome 1, two on chromosome 6, one on chromosome 8, and one on chromosome 10. The loci on chromosome 6 colocalized with the resistance loci discovered by Klein *et al.* (2001) and Mohan *et al.* (2010). Burrell *et al.* (2015) created a RIL population of 117 inbred lines, and generated 619 SNP and three microsatellite markers to create a genetic map for QTL analysis. After phenotyping for anthracnose symptoms, they identified a QTL on chromosome 5 that colocalized with the QTL identified by Cuevas *et al.* (2014) and Perumal *et al.* (2009). The results from these studies, summarized in Table 1, clearly indicate that there is no single source of anthracnose resistance in sorghum. Moreover, these sources of resistance were not in germplasm adapted to the southeastern United States. As a consequence, heritable anthracnose resistance needs to be identified in germplasm suitable for southeastern USA in order to enable successful commercial production of sorghum in this region. The small number of QTL identified in previous studies indicates that, as with many other fungal resistance sources, there are likely dominant resistance loci that can be identified with relatively small-scale studies.

**Table 1:** Reports of QTL for anthracnose resistance organized by chromosomal location

Publication	Chrom.	Distance on Chrom.	Colocalization of Resistance	Method	Markers	Population	Resistance Source	Susceptible Source	Locations*^a^*
Upadhyaya *et al.* 2013	1	9.6 Mb, 25-25.2 Mb, 50.8 Mb, 72.1-72.2 Mb	Tobacco mosaic virus	Association	14,739 SNP	Minicore			1-India
Mohan *et al.* 2010	4	0-8 cM		QTL	149 microsatellite	168 F_7_ RIL	296B	IS18551	2-India
Cuevas *et al.* 2014	5	55-56.1 Mb		BSA	61 SSR	50 F_6_ RIL	SC112-14	PI 609251	1-Puerto Rico, 1-Texas, 1-Arkansas
Perumal *et al.* 2009	5	3.6-4.9 cM from 60.9 Mb		BSA	98 AFLP	71 F_2:3_ families	SC748-5	BTx623	1-Texas, 1-Georgia
Burrell *et al.* 2015	5	53.80– 62.15 Mb		QTL	619 SNP, 3 microsatellite	117 F5 RIL	SC748-5	BTx623	3-Texas, 1-Georgia
Mohan *et al.* 2010	6	0-6 cM, 36-49 cM	Rust, zonate leaf spot, target leaf spot, leaf blight	QTL	149 microsatellite	168 F_7_ RIL	296B	IS18551	2-India
Klein *et al.* 2001	6	94-97 cM	Grain mold, zonate leaf spot, bacterial leaf stripe	QTL	157 AFLP	125 F_5_ RIL	Sureño	RTx430	4-Texas
Upadhyaya *et al.* 2013	6	52.6-52.7 Mb, 56.7 Mb	Rust, zonate leaf spot, target leaf spot, leaf blight, grain mold, bacterial leaf stripe	Association	14,739 SNP	Minicore			1-India
Singh *et al.* 2006	8	3.26 cM, 6.03 cM		BSA	46 RAPD	49 F_8_ RIL	HC136	G73	Greenhouse-India
Upadhyaya *et al.* 2013	8	4.2-4.3 Mb	Bacterial speck	Association	14,739 SNP	Minicore			1-India
Biruma *et al.* 2012	9	4.9 Mb, 56.5 Mb	Rust	RNA-seq		2 inbreds	BS04/05	MU07/193D	Growth chamber-Iowa
Upadhyaya *et al.* 2013	10	48.1-48.6 Mb	Rice blast	Association	14,739 SNP	Minicore			1-India

a Location numbers are representative of independent testing locations, which may be observed over multiple years.

With the exception of Upadhyaya *et al.* (2013), who used the sorghum minicore collection instead of a biparental mapping population, these mapping studies used a relatively small number of genetic markers. With next-generation sequencing technology, a much larger number of genetic markers can be generated for the task of mapping traits of interest within mapping populations, including anthracnose resistance. Genotyping by sequencing (GBS) was designed as a method to generate sequence data for full genomes in a simple, yet robust, method (Elshire *et al.* 2011). This method can be used to generate many SNP markers across a genome without the use of a SNP array. Although the GBS technology is fairly recent, it has been used to generate molecular markers in a number of studies (Atwell *et al.* 2010; Poland and Rife 2012; Spindel *et al.* 2013; Burton *et al.* 2014; Glaubitz *et al.* 2014), with the first reported use in sorghum in 2011 (Nelson *et al.* 2011).

In the present study, a mapping population was developed from a parent line (‘Bk7’) developed at the University of Florida that is highly resistant to *C. sublineola*. The population was grown in three environments in two different years in Florida, and genetic markers were obtained using GBS followed by a custom filtering procedure. Association analysis was performed to determine linkage between the genetic markers and the disease resistance derived from the resistant parent.

## Materials and Methods

### Population development

Sorghum is a diploid, monoecious species with hermaphrodite flowers that produces seed via self-pollination. A biparental mapping population was generated from an initial cross of the parents ‘Bk7’ (female parent; made male-sterile by tying a plastic bag over the flower at the onset of anther dehiscence), and ‘Early Hegari-Sart’ (EH-S). ‘Bk7’ is a grain sorghum generated by Dr. Daniel Gorbet (University of Florida, retired) based on an initial selection made from the genetically diverse GPP5BR germplasm population of *Sorghum bicolor* (L.) Moench (Duncan *et al.* 1991). The pure line ‘Bk7’ resulted from inbreeding the initial selection for nine generations while selecting for resistance to several diseases prevalent in North Florida, most notably anthracnose, and short stature to enable combine harvesting of the seed. EH-S is an experimental forage sorghum line that has been used as a parent for the creation of forage *brown midrib12* (*bmr12*) and male-sterile (*A3*) isolines (Pedersen *et al.* 1982, 2006; Oliver *et al.* 2005), and is susceptible to anthracnose. In addition to the clear contrast in the susceptibility to anthracnose (Figure 1), the parent lines were also selected based on their similarities in stature and maturity. The mapping population consisted of 28 F_3_-derived F_4_ lines (denoted as F_3:4_), and 107 F_4:5_ lines, resulting in a total of 135 lines. The population was generated by self-pollinating all F_1_ and F_2_ plants, random selection of a single plant from each of the resulting F_3_ families for self-pollination, and random selection and self-pollination of a single plant from the resulting F_4_ families. Supplemental Material, Figure S1 illustrates the population development. Since the population was developed in an environment where anthracnose was endemic, this procedure carries a risk of bias against severely infected plants with poor seed set. The susceptible parent EH-S does, however, produce viable seed in this environment. Of the 107 F_4:5_ lines, 24 were derived from a cross of ‘Bk7’ with the EH-S *bmr12* isoline (Pedersen *et al.* 2006). This isoline was used due to a limited number of available wild-type EH-S plants at the time the initial crosses were generated, caused by environmental constraints (*i.e.*, damage from wind, insects).

### Experiment and field layout

The parents and mapping population were planted at the University of Florida Suwannee Valley Agricultural Extension and Education Center in Live Oak, FL (30.313277 N, 82.902158 W), and the Plant Science Research and Education Center in Citra, FL (29.410629 N, 82.170081 W), in the spring of 2013, and at the Live Oak, FL location in the summer of 2015. In 2015, a subset of 92 lines of the original F_4_ generation were planted, rather than the F_3:4_/F_4:5_ progeny, due to limited seed availability. The field experiments were laid out in a randomized complete block design population with two replications at Live Oak in both 2013 and 2015, and three replications at Citra in 2013. The field plots were 4.6 m long, and spaced 0.76 m apart. The pre-emergence herbicide Dual Magnum (Syngenta, Research Triangle Park, NC) was applied prior to planting in conjunction with application of Concep II safener (Syngenta, Research Triangle Park, NC) to the seeds using application rates recommended by the manufacturers. Rolling cultivation was performed on the fields in the early stages of growth, followed by sweep cultivation at the five-leaf stage. Irrigation was provided as a supplement to natural rainfall, and was applied by a pivot irrigator at Live Oak, and a lateral moving irrigator at Citra. The fields were fertilized with 250 kg ha^–1^ of NPK 10-34-0 before planting, and supplemental fertilizers were applied as needed through fertigation. Insecticides were used as needed to limit the damage from head worms, aphids, or other pests. Due to the endemic presence of *C. sublineola* at these sites, all evaluations were based on natural infection with the pathogen.

### Phenotyping

For the phenotyping of anthracnose resistance, each row was scored for disease severity by assessing multiple plants within the row and assigning a row score. The disease severity was scored on a scale of 1 to 5 (Table S1), following the field screening method by Thakur *et al.* (2007): 1, representing no visible symptoms or small flecks; 2, less than ∼10% of the leaf area covered with dark lesions; 3, ∼10–25% of the leaf area covered with dark or necrotic lesions; 4, ∼25–50% of the leaf area covered with dark or necrotic lesions; and 5, more than ∼50% of the leaf area covered with dark or necrotic lesions. Since maturity has been reported to affect the appearance of anthracnose symptoms (Ferreira and Warren 1982; Ashok *et al.* 1992; Peacocke 1995), disease scores were taken over the course of 5 wk, when individual lines were at soft-dough seed developmental stage. All measurements were taken by a single rater (T.F.).

**Figure 2:**
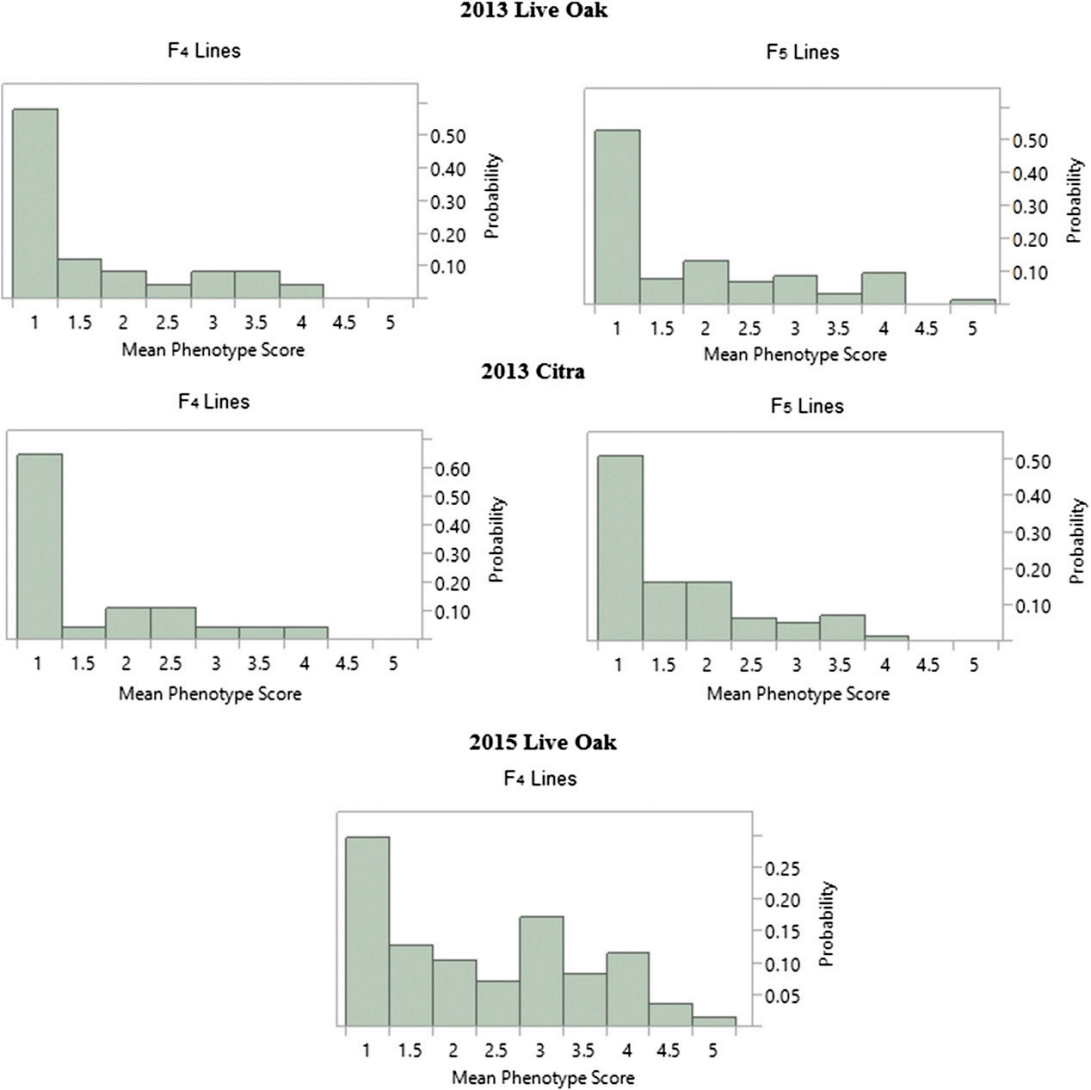
Histograms of the averaged phenotypic disease score. Histograms were generated for each location, inbred generation (F_4_ and F_5_) and year.

Even though the anthracnose severity was scored on a five-point scale, the majority (∼80% for 2013, ∼60% for 2015) of the lines received a score of 1 or 2 (Figure 2). This skew indicated resistance, when present, is strong. Accordingly, the phenotypic data were transformed into a more concise resistant/susceptible scale. Scores were combined across replicates so that consistent scores of 1 or 2 across were considered resistant (0), while consistent scores of 4 or 5 across reps were considered susceptible (2), and scores that varied between clearly resistant and clearly susceptible across reps were scored as ambiguous (1). Other transformations were performed and all gave comparable results (Table S2). Plant height was recorded for all lines based on the average of three plants as the distance between the ground and the top of the panicle.

### Tissue collection and genotyping

Leaves were collected from five different plants per line, and stored frozen in a –80° freezer. Samples were collected in both 2013 and 2015 because they represented different generations, F_3:4_/F_4:5_ and F_4_, respectively. DNA was extracted from the pooled, frozen ground tissue samples with the Qiagen DNeasy Plant DNA Extraction kit (Germantown, MD). The DNA was sent to the Institute of Biotechnology at Cornell University (Ithaca, NY) for GBS as described by Elshire *et al.* (2011). Samples whose reads accounted for less than 10% of the mean sequenced reads in the sequencing lane were removed because this indicated failed sequencing reactions (Elshire *et al.* 2011). There were only four samples with fewer than 20,000 reads. Since these samples did not yield enough data to generate SNPs, they were eliminated from further consideration. Additionally, a total number of 300,000,000 high-quality reads per lane had to be generated in order to include the data from a given lane. SNPs were derived from the sequencing data by aligning the reads to the *Sorghum bicolor* reference genome v2.4 (Paterson *et al.* 2009) and identifying SNPs from the aligned reads using the TASSEL 3.0 GBS pipeline (Glaubitz *et al.* 2014). During alignment of the reads, tags were created from the reads with the following parameters: tags needed to have at least three instances, average sequencing error rate per base less than 1%, and conflicting genotype calls were called as heterozygous (Glaubitz *et al.* 2014). The tags were subsequently aligned to the sorghum reference genome v2.4 using Burrows-Wheeler aligner with default values (Li and Durbin 2010). Tags that did not map uniquely were discarded. Tags with unique positions were used to identify 130,569 SNPs in the F_3:4_/F_4:5_ and 101,075 in F_4_ populations.

The tag filtering does not remove nonpolymorphic SNP markers, markers with high levels of heterozygosity, or missing data. A custom filtering procedure was developed (Figure S2 and File S1). Starting with the 130,569 and 101,075 SNPs, all markers for which the parents’ alleles were identical (not polymorphic), or for which one parent was missing and the other parent was heterozygous (ambiguous genotypes), were removed. Furthermore, markers were removed when they had a minor allele frequency (MAF) of ≤10%, had > 80% heterozygosity, or an inbred heterozygosity score (panmixia) of < 0.8, because these indicate likely errors in the sequencing results (Elshire *et al.* 2011; Sim *et al.* 2012; Morris *et al.* 2013; Glaubitz *et al.* 2014; Liu *et al.* 2014; Swarts *et al.* 2014). These parameters resulted in a more stringent filtering than the default TASSEL-based filtering procedure applied at Cornell University, which by default removes markers with MAF ≤1%, ≥80% missing data, and markers with more than two allele calls. In this population, these less stringent defaults retained highly heterozygous markers, and markers with heavily skewed allelic distributions, resulting in markers for which too few lines remained for proper statistical evaluation. In addition, closely linked markers that provided identical genotypic information across the population were removed to reduce redundancy in the data; in these cases every marker removed was within 65 bp from the marker that was retained. The custom filtering procedures reduced the number of informative SNP markers to 5186 for the F_3:4_/F_4:5_ data, and 2759 for the F_4_ data.

In order to further improve the quality of the map, missing marker data were imputed using the length imputation procedure in TASSEL 3.0 (Bradbury *et al.* 2007), with a window size of 10 and an allowed mismatch of 1 (Atwell *et al.* 2010; Spindel *et al.* 2013). The imputed and original SNPs were converted to a biparental naming system (*i.e.*, a SNP derived from EH-S was labeled as “a”, a SNP derived from ‘Bk7’ was labeled as “b”, and heterozygotes were labeled as “h”). Visual inspection of the marker data in their chromosomal positions showed the presence of double recombinations in areas where such an event was unlikely, and multiple double recombination events inside a wide region containing the alternate allele in the flanking regions on both sides. The recombinations between consecutive markers were counted across the entire genome. If one marker within a six-marker window indicated a potential double recombination, this was assumed to be a sequencing-based error and that marker/line combination was adjusted to the allele of the flanking markers. If three or more recombinations occurred within a sliding, four-marker window, all four markers within the window were treated as missing for that line. In the 24 lines derived from the EH-S-*bmr12* parent, the area flanking the *bmr12* gene (chromosome 7; 1.9 Mb–7.1 Mb), along with regions in chromosomes 8, 9, and 10, were treated as missing. These were genomic regions derived from the original *bmr12* donor (Pedersen *et al.* 2006), and GBS calls were more variable than in the remainder of the genome. Markers were imputed with TASSEL 3.0, with the same settings as above, for all regions except those associated with the *bmr12* parent. The filtered marker list is available as File S2 and File S3.

### Genetic mapping

JoinMap 4.1 (Van Ooijen 2006) was used to generate a recombination-based map in this population. The markers were sorted by the percent data missing in descending order (Russell *et al.* 2014), and imported into JoinMap. Genetic linkage groupings were generated in JoinMap by separating the markers into groups using a 1 LOD increment from 2 to 20 LOD, and the resulting genetic linkage groupings were used to generate the chromosomes for the linkage map. The linkage map was generated by the Maximum Likelihood method in JoinMap, with the parameters effectively increased two-fold due to the fact that the default values are designed for marker groups of about 100 markers (Van Ooijen 2006), and the groups generated for this project contained at least 200 markers. The parameters changed were: number of map optimization rounds per sample (10), map order chain length (2000), cooling control parameter (0.0005), number of nonimproved chain cut-off (20,000), length of burn-in chain (20,000), number of Monte Carlo cycles (8), multipoint estimation chain length (2000), and sampling period for matrix samples (10).

### Association analysis

An association analysis was conducted to identify markers linked to disease resistance using the two-tailed Fisher’s exact test (Fisher 1922) following removal of lines with inconsistent (ambiguous) resistance scores. Full results are available as File S3 and File S4. Assumption for the Fisher’s exact test were met, as there were no tables where zero counts were observed, and lines are independent. For this analysis, heterozygotes were treated as missing values, although QTL results were consistent with other approaches to the analysis of heterozygotes (Table S3 and Table S4). The threshold for determining a significant association between the phenotypic and genotypic data were determined with a false discovery rate (FDR) with α= 0.05 (Benjamini and Hochberg 1995; reviewed in Verhoeven *et al.* (2005)). To determine which parent contributed the resistance, an association analysis was performed using the left-tailed and right-tailed Fisher’s exact test. The left-tailed Fisher’s exact test identifies associations between ‘Bk7’-derived alleles and resistance, whereas the right-tailed Fisher’s exact test identifies associations between EH-S-derived alleles and resistance.

After the QTL were identified, the genotype of the QTL for each line of the populations (F_4:5_, F_3:4_, or F_4_) was recorded based on a greater than two-third majority of markers in the region. To estimate the proportion of variance attributed to that QTL, a logistic regression was used and the proportion of variance estimated as the generalized *r*-square (*c*^2^). Lines with ambiguous QTL in a particular region were removed before estimation.

### Similarity search

Transcripts within the identified resistance loci were obtained from Phytozome 10.2 (Goodstein *et al.* 2012), and were derived from the annotated sorghum reference genome v2.4 (Paterson *et al.* 2009). Sequence similarity searches of the sorghum transcripts were conducted using BLASTX Command Line (Gish and States 1993) with the following settings: no alignments kept (-num_alignments 0), only hits with expect values greater than 40 saved (-evalue 40), use 8 threads for search (-num_threads 8), and export data as XML file (-outfmt 5). The BLASTX results were annotated for gene ontology groups with the BLAST2GO Pipeline (Conesa *et al.* 2005) using the default properties. The annotation files were imported into the BLAST2GO GUI and enrichment tests were performed.

To assess the possibility of *R*-genes outside the annotated sorghum transcripts, a multifasta file containing all curated reference *R*-genes was downloaded from the Plant Resistance Gene Database (Sanseverino *et al.* 2013). These reference *R*-genes were used in BLAST with default settings to identify similar sequences in the sorghum genome (v2.4). The genes identified as defense-related genes within the QTL were placed along the sorghum reference genome sequence (v2.4) to identify potential gene clusters.

### Validation experiment

An independent validation experiment was conducted using a set of four recently released cultivars derived from a cross between ‘Bk7’ and the sweet sorghum ‘Mer81-4’. The cultivars were generated using the pedigree method (Hayes and Johnson 1939), and released by the University of Florida after six generations of inbreeding and selection (Table S5). Anthracnose resistance (originating from ‘Bk7’) was one of the main selection criteria. SNPs identified in the GBS data were used to design primers that enabled allele-specific PCR within the resistance locus on chromosome 9 (Table S5). Genomic DNA was extracted from seedlings germinated in a growth chamber using the Qiagen DNeasy Plant DNA Extraction kit (Germantown, MD). PCR products were generated using Red*Taq* ReadyMix (Sigma-Aldrich, St. Louis, MO), containing 30 ng genomic DNA, and 0.2 µM of each primer using the PCR programs listed in Table S5 on a C-1000 Touch thermal cycler (BioRad, Hercules, CA). PCR products were analyzed on 2% (w/v) agarose gels containing GelRed (Biotium, Inc., Hayward, CA) and the resulting data were used to genotype all four resistant cultivars.

### Data availability

All of the original data described in this manuscript are available as supplemental files, tables and figures (Table S1, Table S2, Table S3, Table S4, Table S5, Table S6, Table S7, Table S8, Table S9, Figure S1, Figure S2, Figure S3, Figure S4, Figure S5, Figure S6, File S1, File S2, File S3, File S4, File S5, File S6, File S7, and File S8). The file named ‘Felderhoff Supplemental Text’ contains the legends for the Supplemental Tables Figures, and Files. Supplemental files include the SAS code to filter the GBS-derived SNP markers (File S1), the list of filtered markers for both GBS experiments (File S2 and File S3), the results from Fisher’s exact test for both GBS experiments (File S4 and File S5), the original GBS data in hapmat format for both experiments (File S6 and File S7), and a list of markers with identical GBS genotypic data (File S8).

## Results and Discussion

### Phenotypic analysis suggests anthracnose resistance is inherited in a dominant fashion

The F_1_ progeny derived from the cross of ‘Bk7’ and EH-S were resistant to anthracnose and were similar in height and maturity to the two parents. The F_2_ population showed variation in both height (Figure S3) and maturity consistent with a complex multigene segregation, with a high proportion of the population resistant to anthracnose.

These initial observations are consistent with the findings of other studies in sorghum and closely related species, where resistance patterns are binary in nature. Since genomic regions associated with anthracnose resistance have been identified with smaller populations and fewer inbreeding generations (Klein *et al.* 2001; Mignouna *et al.* 2002; Voorrips *et al.* 2004; Burrell *et al.* 2015), we genotyped and phenotyped our population at the F_4_ and F_3:4_/F_4:5_ generations with the goal of broadly identifying the source of resistance in ‘Bk7’. This approach offers the benefit that when small numbers of loci are expected, time and resources can be efficiently directed on advanced studies to identify specific genes (Melotto and Kelly 2001; Cuevas *et al.* 2014), especially given the high-density of markers generated by GBS. Histograms of the phenotypic disease scores for all environments are displayed in Figure 2. The observations that the F_1_ was resistant and that a high proportion of lines in the mapping population were resistant were considered an indication that anthracnose resistance derived from ‘Bk7’ followed the pattern for disease resistance observed in other species, and therefore a justification for the adoption of an early mapping strategy.

### An improved genetic map based on filtered GBS data

**Figure 3:**
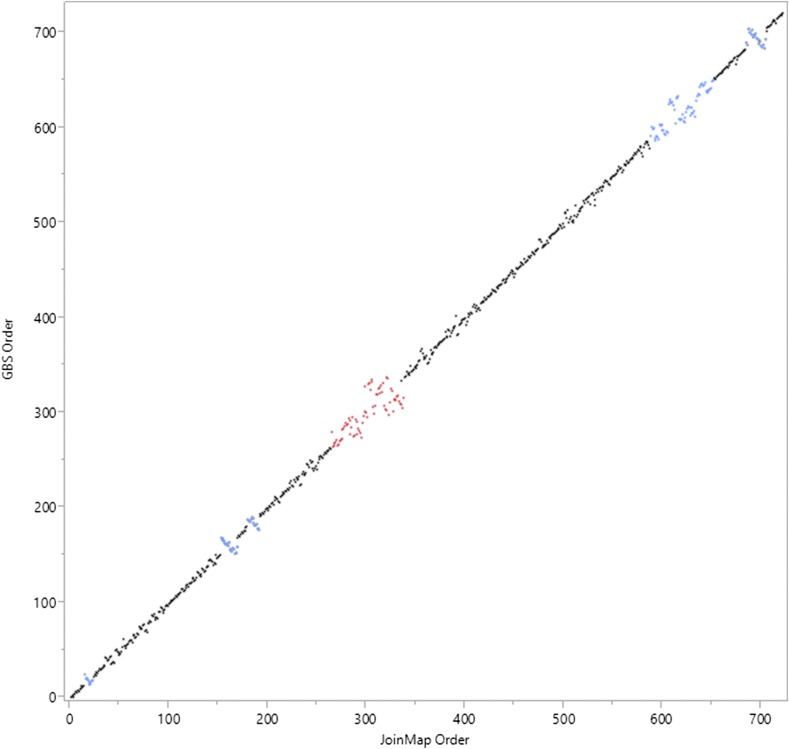
Bivariate comparison between the marker orders according to the reference genome and JoinMap for chromosome 1 following custom filtering. The *x*-axis represents the order according to the linkage map generated by JoinMap, the *y*-axis represents the order according to the reference genome v2.4 (GBS ordering). When the slope of the data points is equal to one, the two methods are in agreement on the marker order. Points that do not fall on the line reflect markers that differ in order depending on the method used. The red points represent the centromere and the blue points mark an area of high marker density.

Initial development of a genetic map using JoinMap based on a set of 5186 markers showed several regions that were inconsistent with the marker order based on the reference genome (Figure S4). There are two plausible explanations for discordance: genome rearrangements between the parents of the population and the reference line (inbred BTx623; Paterson *et al.* 2009), or poor marker quality combined with poorly resolved and tightly linked markers. After filtering GBS markers for potentially poor quality calls, concordance between JoinMap and genome reference was excellent (Figure 3). The few areas where the JoinMap order differed from the reference genome order was over the centromeres, which is likely due to large areas with little recombination, and positions of high marker density in a short map interval, making it difficult to order these closely linked markers based on recombination events. We conclude that there is no substantial evidence for genome rearrangements between ‘Bk7’ and EH-S relative to ‘BTx623’.

### Association analysis identifies two independent resistance loci derived from ‘Bk7’

**Figure 4:**
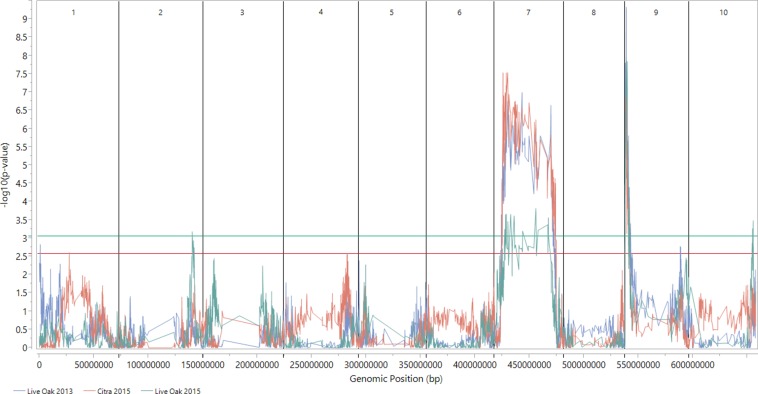
Association map of anthracnose resistance in the mapping population phenotyped in three environments. The graph shows the degree of association between the markers and the disease phenotype. The *x*-axis represents the genomic position, and the *y*-axis represents the –log_10_(p-value) obtained from Fisher’s exact test. A higher *y*-value indicates a stronger association between the marker at that position and disease resistance. The blue, red, and green lines represent the environments of Live Oak 2013, Citra 2013, and Live Oak 2015, respectively. The vertical black lines separate the 10 chromosomes, while the horizontal lines mark the FDR significance thresholds of 5%, with the colors corresponding to the environments; the FDR-lines for the two 2013 environments are indistinguishable from one another in this figure.

We identified a large QTL on chromosome 7 and a smaller one on chromosome 9 (Figure 4, Table S3, and Table S4). These results were consistent across years and environments, and not sensitive to the details of analysis choices (Figure S5 and Figure S6).

The locus on chromosome 7 includes the centromere, and spans 48.7 Mb based on the F_3:4_/F_4:5_ data, whereas the locus on chromosome 9, located at the distal end of the short arm, covers 4.5 Mb or 3.2 Mb, based on the F_3:4_/F_4:5_ and F_4_ data, respectively (Figure 4). The large map interval on chromosome 7 may be due to low recombination around the centromeric region, or a high degree of linkage disequilibrium, possibly because this chromosome harbors several loci that were relevant in the domestication of sorghum (Morris *et al.* 2013). There have been no prior reports in the literature for QTL or genes linked to anthracnose resistance on chromosome 7, making this a novel finding. The *Cs2A* and *Cs2B* resistance genes identified by Biruma *et al.* (2012) map to the distal end of the long and short arms of chromosome 9, respectively. The distance between the closest edge of the resistance locus identified in this study and *Cs2B* is 1.8 Mb. There are several recombination events between the QTL and the known *Cs2B* resistance gene, providing evidence that the source of ‘Bk7’ resistance on chromosome 9 is also novel.

**Figure 5:**
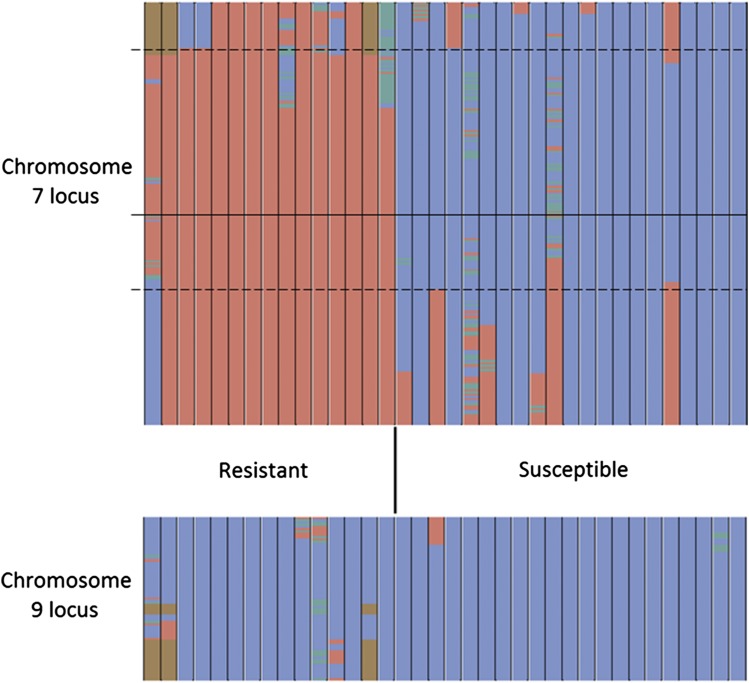
Genotypic markers within the two QTL for individual lines organized by phenotype. The vertical columns represent individual lines, and the color within the columns indicate the genotypic state of the markers: blue represents ‘Early Hegari-Sart’, red represents ‘Bk7’, green represents heterozygosity, and brown represents missing data. The scale on the *y*-axis is proportional to the number of markers. The reduced QTL region is indicated by the dashed black lines, while the solid black line indicates the position of the centromere on chromosome 7. Lines with ambiguous phenotypic data were excluded for clarity.

To visualize the effects of the QTL, lines were separated into categories based on their marker genotypes within the identified QTL. The large QTL on chromosome 7 was divided in two sections encompassing the markers located on the short arm and long arm, respectively. This nominal division was performed due to the large size of, and inclusion of the centromere within, the QTL. The corresponding phenotypic values are reported (Table 2 and Table S6). The most susceptible lines contain EH-S alleles at all of the QTL, whereas the most resistant lines contain ‘Bk7’ alleles at all of the QTL. The proportion of phenotypic variation attributable to the presence of resistance loci was calculated individually for each locus (Table 3). The maximum proportion of phenotypic variation explained by ‘Bk7’-derived loci on chromosome 7 short and long arms and chromosome 9 was 0.56, 0.65, and 0.70, respectively. Comparing the recombination between the resistant and susceptible lines, a region of minimal overlap was determined and is reported as the final QTL interval for chromosome 7 (Figure 5).

**Table 2:** Distribution of phenotypes based on the parental origin of the marker alleles

	Parental Allele				Phenotype		
Population	Chr7Short	Chr7Long	Chr9	Total	Resistant	Ambiguous	Susceptible
F_4:5_	BB	BB	BB	9	8	1	
F_3:4_	BB	BB	BB	5	5		
F_4_	BB	BB	BB	11	10	1	
F_4:5_	BB	BB	EE	15	13	2	
F_3:4_	BB	BB	EE	1	1		
F_4_	BB	BB	EE	9	4	4	1
F_4:5_	BB	EE	BB	4	4		
F_3:4_	BB	EE	BB	1	1		
F_4_	BB	EE	BB	1	1		
F_4:5_	EE	BB	BB	2	2		
F_3:4_	EE	BB	BB	2	2		
F_4_	EE	BB	BB	1	1		
F_4:5_	BB	EE	EE	2	1		1
F_4_	BB	EE	EE	1		1	
F_4:5_	EE	BB	EE	7	2	1	4
F_4_	EE	BB	EE	1	1		
F_4:5_	EE	EE	BB	18	17	1	
F_3:4_	EE	EE	BB	2	1	1	
F_4_	EE	EE	BB	14	12	2	
F_4:5_	EE	EE	EE	17		6	11
F_3:4_	EE	EE	EE	4		1	3
F_4_	EE	EE	EE	19		2	17

QTL for each line were classified as either derived from Bk7 (BB) or derived from Early Hegari-Sart (EE). The genotypes are separated by generation (Population). Due to its large size, the QTL on chromosome 7 was separated into two segments, on the short arm (Chr7Short) or the long arm (Chr7Long).

**Table 3:** Proportion of phenotypic variation attributable to the identified QTL based on a logistic regression analysis between the predicted and actual resistance phenotypes

Location	Locus	Variation Explained (*c*^2^)
F_4:5_	Chr7 short arm	0.19
F_4:5_	Chr7 long arm	0.19
F_4:5_	Chr9	0.51
F_3:4_	Chr7 short arm	0.35
F_3:4_	Chr7 long arm	0.12
F_3:4_	Chr9	0.52
F_4_	Chr7 short arm	0.17
F_4_	Chr7 long arm	0.25
F_4_	Chr9	0.54

### The resistance loci contain candidate defense-related genes, some of which are present in clusters

The QTL on chromosomes 7 and 9 contain 589 and 342 genes, respectively. Analysis of the genes within these two resistance loci via similarity search using BLASTX revealed a number of candidate genes that can be responsible for the observed resistance against *C. sublineola*. As summarized in Table S7, the sequence of 40 genes within the resistance locus on chromosome 7 and 36 genes within the locus on chromosome 9 show similarity to genes associated with disease resistance in general. Among these candidate genes are genes encoding NBS-LRR-containing proteins (as discussed before), chitinases, germin-like proteins, polyphenol oxidases, peroxidases, ABC-transporters, and defensins. Genes encoding chitinases are effective at attacking pathogenic fungi (reviewed by Grover 2012), while the expression of genes encoding germin-like proteins (Manosalva *et al.* 2009), polyphenol oxidases (Constabel and Barbehenn 2008), and peroxidases (Almagro *et al.* 2009) result in the strengthening the plant cell wall, making it more difficult for invading pathogens to penetrate. Genes encoding ABC-transporters may be involved in transporting toxins across the plasma membrane at the sites of attempted pathogen invasion (Stein *et al.* 2006), while defensins are small, basic peptides with antifungal activity (reviewed by Thomma *et al.* 2002). This led us to hypothesize that these QTL contain clusters of defense-related genes.

To further evaluate the possibility of *R*-gene orthologs present within the QTL, the curated reference list of *R*-genes created by the Plant Resistance Gene Database was aligned to the sorghum reference genome v2.4 using BLASTN. The BLAST search identified 60 sequences with similarity to the reference *R*-genes (Table S8) within the two loci: 22 on chromosome 7, and 38 on chromosome 9. A total of 39 aligned with putative resistance gene transcripts, while eight transcripts with similarity to *R*-genes had not been annotated as putative resistance genes. The remaining 13 sequences, matching eight individual *R*-genes, showed similarity to sorghum genomic sequences that were not annotated as expressed genes (Table S9). The putative resistance genes identified in the BLAST2GO search, along with any of these 13 sequence matches that turn out to represent expressed sorghum genes are considered candidate anthracnose resistance genes.

**Figure 6:**
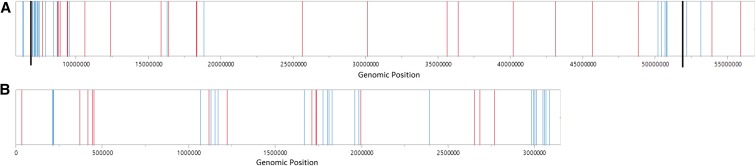
Genomic positions of candidate resistance genes within the two resistance loci. The bars represent the starting location for the annotated transcript of the candidate genes on (A) the locus on chromosome 7, and (B) the locus on chromosome 9. A red bar indicates an *R*-gene while a blue bar indicates a defense-related gene that is not an *R*-gene. The thick vertical black lines on the chromosome 7 locus indicate the size reduction of this QTL based on Figure 5. The *x*-axis represents the genomic position within each chromosome. The *x*-axes in A and B differ in scale.

There are several distinct clusters of genes with high sequence similarity to defense-related genes, including clusters of *R*-genes, in the QTL (Figure 6). Clustering of linked *R*-genes is a feature that has been observed in other plants (Botella *et al.* 1998; Kang *et al.* 2011; Zhao *et al.* 2011; Cook *et al.* 2012; McHale *et al.* 2012). The formation of *R*-gene clusters can result from *R*-gene duplication and divergence, thus increasing the range of elicitors that can be detected. Depending on the extent of divergence, this can result in resistance against different isolates of a specific pathogen (Botella *et al.* 1998), or against entirely different species of pathogens (Michelmore and Meyers 1998). In common bean (*Phaseolus vulgaris* L.), where resistance to anthracnose (caused by *C. lindemuthianum*) is known to be derived from several independently inherited dominant and recessive *R*-genes, as well as from clusters of linked *R*-genes (Kelly and Vallejo 2004). Based on the presence of clusters of putative defense-related genes, it is possible that the observed anthracnose resistance in ‘Bk7’ is caused by the interaction of several of these genes.

### Resistant cultivars harbor ‘Bk7’-derived alleles at the resistance locus on chromosome 9

An independent validation experiment was conducted for the ‘Bk7’ resistance locus on chromosome 9. Four newly released anthracnose-resistant sweet sorghum cultivars derived from a cross between ‘Bk7’ and the sweet sorghum ‘Mer81-4’, which had been generated prior to the results of the mapping experiment, were genotyped using SNP markers on chromosome 9 (Table S5). This analysis confirmed the presence of ‘Bk7’ alleles in the center of the QTL in all four cultivars. ‘Mer81-4’-derived marker alleles closer to the distal and proximal ends of the QTL were present in subsets of the four cultivars. This analysis reduced the size of the QTL on chromosome 9 to 506 kb. This is an independent validation of this region as a source of resistance from the ‘Bk7’ parent.

### Conclusions

Identification of novel sources of anthracnose resistance is vital for expanding the production of sorghum in the southeastern United States, and will also be of value in other regions where anthracnose is of concern. Based on an association analysis, segregation data, sequence similarity searches, and gene ontologies, the anthracnose resistance of inbred line ‘Bk7’ appears to be derived from resistance loci on chromosomes 7 and 9 that together explain the majority (> 80%) of the observed phenotypic variation in the mapping population. The simple inheritance pattern of the anthracnose resistance from ‘Bk7’ enabled the use of a relatively small mapping population that was genotyped and phenotyped early, namely at the F_4_ and F_5_ generations. The benefits and drawback of this approach were apparent from the resolution at the two chromosomal locations: a very large QTL on chromosome 7 that contained too many candidate genes for validation studies, and that may in fact represent two separate QTL on opposite sides of the centromere, *vs.* a small QTL on chromosome 9 that could be independently validated in sweet sorghum cultivars that inherited their anthracnose resistance from ‘Bk7’. This validation experiment also reduced the size of the QTL, which now enables fine mapping studies to identify the causal gene(s) behind the anthracnose resistance at this locus. The QTL on chromosome 7 will need to be reduced in size, for example, through continued inbreeding, backcrossing of selected lines from the mapping population to the susceptible parent EH-S, or detailed genetic analysis of the same cultivars used for the validation of the QTL on chromosome 9. Given the presence of clusters of defense-related genes at both loci, the possibility exists that the observed anthracnose resistance is the result of the concerted action of linked genes, which will need to be considered in follow-up validation studies.

## Supplementary Material

Supplemental Material
