# Supplementary material for: Using Genotyping by Sequencing to Map Two Novel Anthracnose Resistance Loci in Sorghum bicolor
Source: G3 (Bethesda). 2016 May 18;6(7):1935–46. doi: 10.1534/g3.116.030510 (PMC4938647; doi:10.1534/g3.116.030510)
Supplement: Supplemental Material [file supp_6_7_1935__index.html]

Using Genotyping by Sequencing to Map Two Novel Anthracnose Resistance Loci in Sorghum bicolor — Supplemental Material 

# Using Genotyping by Sequencing to Map Two Novel Anthracnose Resistance Loci in *Sorghum bicolor*

## Supplemental Material for Felderhoff *et al.*, 2016

**Files in this Data Supplement:**

- Figure S5 - Association analysis overlaying different transformations used for association analysis. (.pdf, 243 KB)
- File S9 - Legends for supplemental material. (.pdf, 486 KB)
- Table S1 - Disease scores for the different lines in the original five-point scale. (.csv, 23 KB)
- Table S2 - Phenotypic scores for the different transformations and locations. (.csv, 4 KB)
- Table S3 - SNP markers associated with anthracnose resistance based on a -log10(p-value) above the FDR significance threshold. (.csv, 14 KB)
- Table S4 - Comparison of the effect on the loci due to the different phenotypic scales and approaches for labeling the heterozygous markers. (.csv, 17 KB)
- Table S5 - Allele-specific PCR to identify 'Bk7'-derived alleles on chromosome 9 in four anthracnose-resistant cultivars. (.csv, 1 KB)
- Table S6 - Complete distribution of phenotypes based on the parental origin of the marker alleles. (.csv, 1 KB)
- Table S7 - Proteins and gene ontology groups identified from the sorghum transcripts within the loci of interest. (.csv, 168 KB)
- Table S8 - Curated reference *R*-genes aligned to the identified anthracnose resistance loci. (.csv, 5 KB)
- Table S9 - Reference *R*-genes with similarity to the sorghum reference genome identified via BLAST. (.csv, 928 B)
- Figure S1 - Visual representation of the development of the mapping population. (.tif, 541 KB)
- Figure S2 - Flowchart for the steps used in the filtering of GBS markers. (.tif, 56 KB)
- Figure S3 - Histograms displaying the distribution of plant height across the population in the three environments. (.tif, 52 KB)
- Figure S4 - Comparison between the marker order on chromosome 7 generated by JoinMap and the marker order based on the reference genome. (.tif, 51 KB)
- Figure S6 - Two-tailed Fisher's exact test for association analysis. (.tif, 176 KB)
- File S1 - SAS code for the 2013 GBS marker filtering steps and Fisher exact test. (.txt, 139 KB)
- File S2 - Final GBS markers from 2013 after all filtering steps are applied. (.csv, 1 MB)
- File S3 - Final GBS markers from 2015 after all filtering steps are applied. (.csv, 570 KB)
- File S4 - The -log10(p-value) of the Fisher's exact test for every marker, location in 2013, phenotypic scale, and heterozygous call. (.csv, 2 MB)
- File S5 - The -log10(p-value) of the Fisher's exact test for every marker, phenotypic scale, and classification of heterozygous markers for the Live Oak 2015 location. (.csv, 493 KB)
- File S6 - Original GBS data from 2013 in hapmat format. (.txt, 40 MB)
- File S7 - Original GBS data from 2015 in hapmat format. (.txt, 23 MB)
- File S8 - List of markers with identical GBS genotypic data. (.txt, 23 KB)
