# Supplementary figures and images for: Using Genotyping by Sequencing to Map Two Novel Anthracnose Resistance Loci in Sorghum bicolor
Source: G3 (Bethesda). 2016 May 18;6(7):1935–46. doi: 10.1534/g3.116.030510 (PMC4938647; doi:10.1534/g3.116.030510)

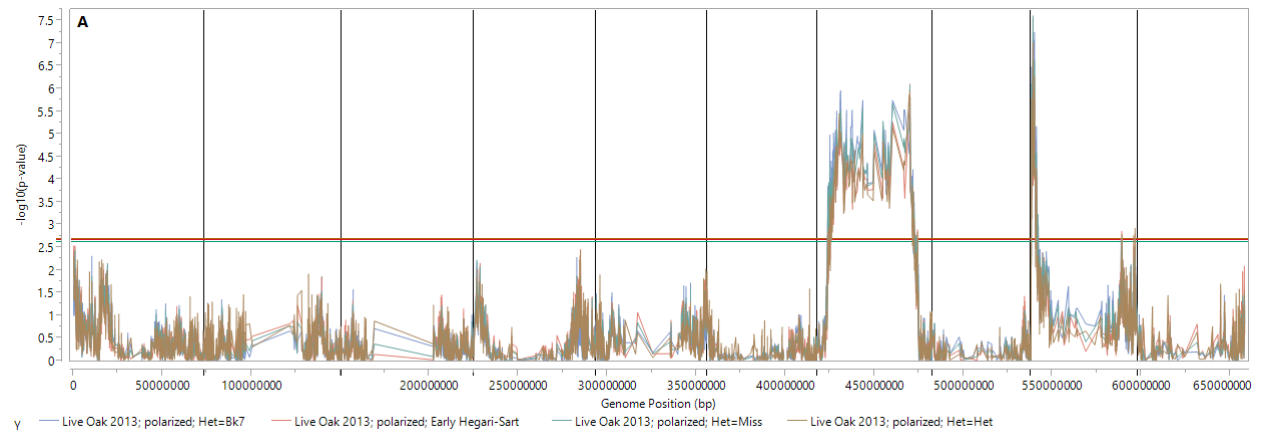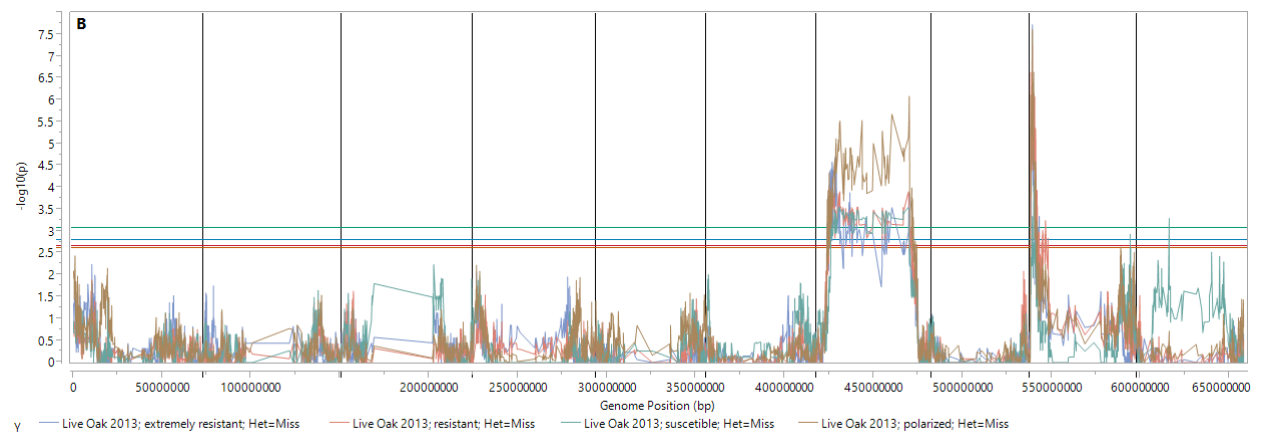

Supplement: Supplemental Material [file supp_g3.116.030510_FigureS5.pdf]

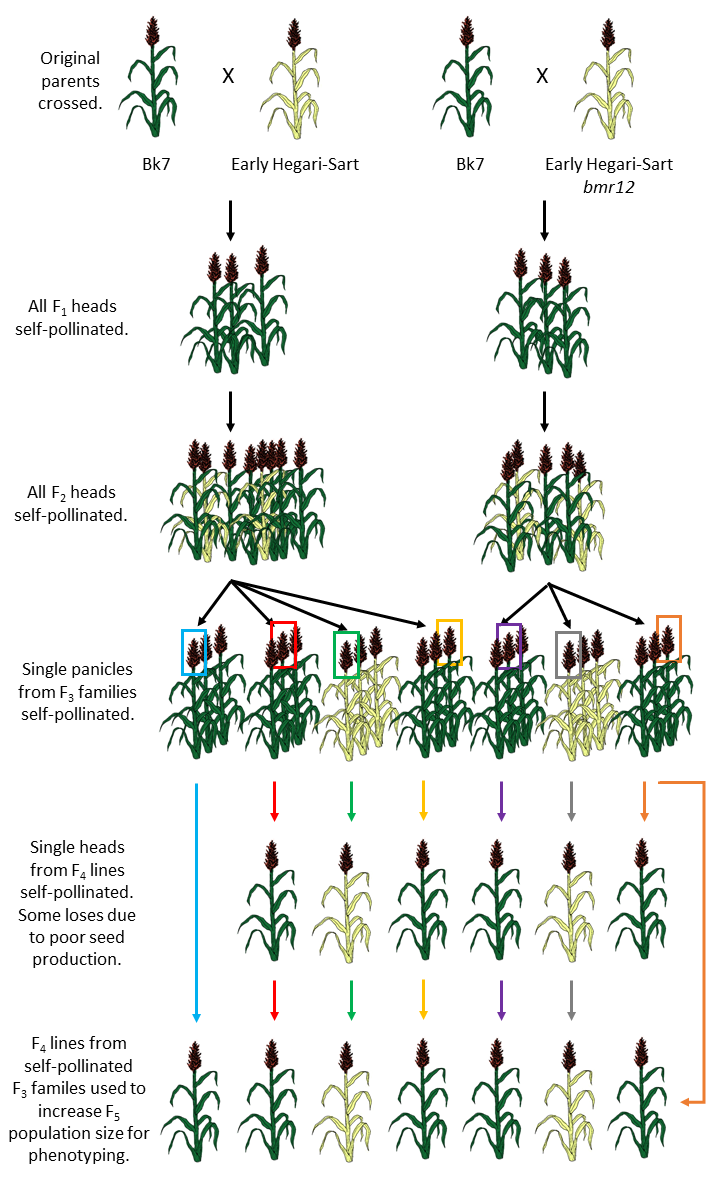

Supplement: Supplemental Material [file supp_g3.116.030510_FigureS1.tif]

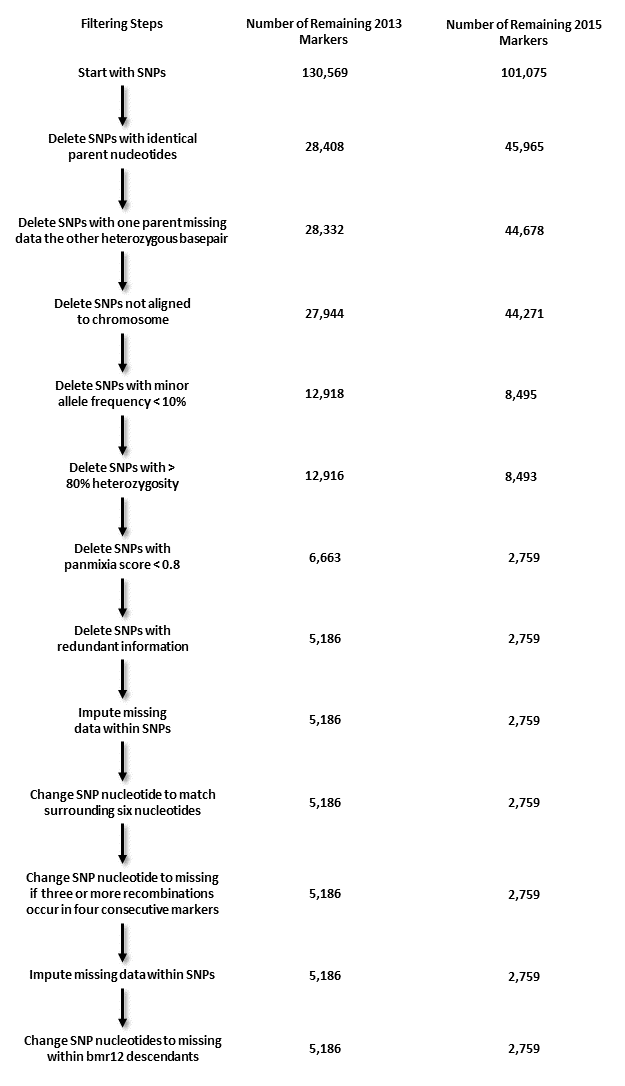

Supplement: Supplemental Material [file supp_g3.116.030510_FigureS2.tif]

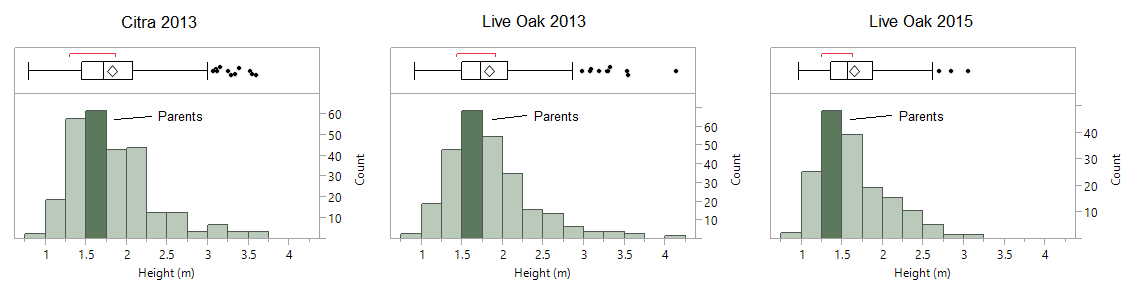

Supplement: Supplemental Material [file supp_g3.116.030510_FigureS3.tif]

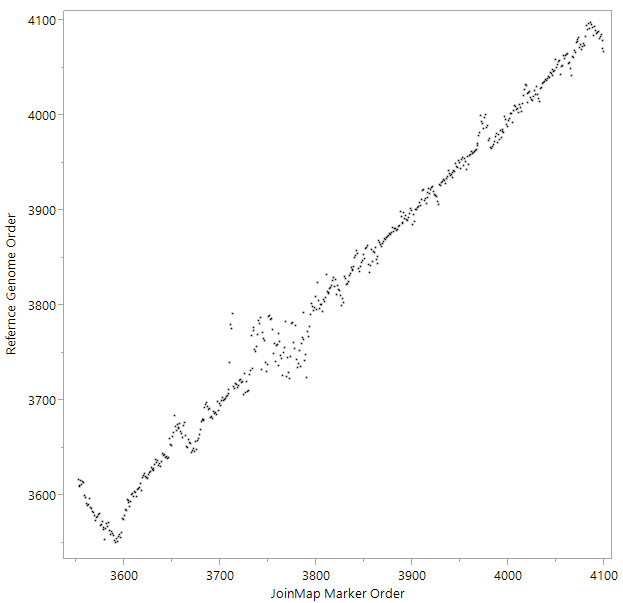

Supplement: Supplemental Material [file supp_g3.116.030510_FigureS4.tif]

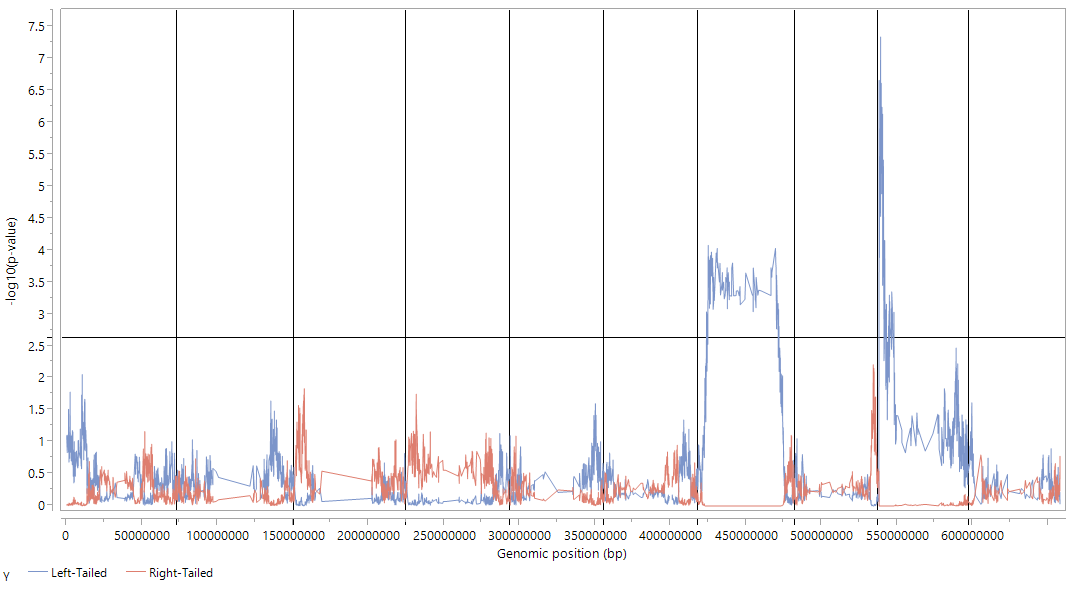

Supplement: Supplemental Material [file supp_g3.116.030510_FigureS6.tif]
