# Supplementary material for: Using Genotyping by Sequencing to Map Two Novel Anthracnose Resistance Loci in Sorghum bicolor
Source: G3 (Bethesda). 2016 May 18;6(7):1935–46. doi: 10.1534/g3.116.030510 (PMC4938647; doi:10.1534/g3.116.030510)
Supplement: Supplemental Material [file supp_g3.116.030510_FileS9.pdf]

Table S1 Disease scores for the different lines in the original five-point scale. The data are in a stacked format. Column headers are in parentheses. Each row represents the original five point phenotypic scale (Disease_Score) with the F_4 or F_5 line (Line), the generation (Generation), the location (Location), and the replication number within location (Rep).

Table S2 Phenotypic scores for the different transformations and locations. Column headers are in parentheses. Each row represents an individual genotype (Line), with individual columns for each transformation (Live_Oak_2013_Extremely_Resistant, Live_Oak_2013_Resistant, Live_Oak_2013_Susceptible, Live_Oak_2013_Polarized, Citra_Extremely_Resistant, Citra_Resistant, Citra_Susceptible, Citra_Polarized, Live_Oak_2015_Extremely_Resistant, Live_Oak_2015_Resistant, Live_Oak_2015_Susceptible, Live_Oak_2015_Polarized). For the 'extremely resistant scale', scores were combined so that all lines with an average of ≤1.33 across replicates were given a score 0 (resistant), and all other lines were scored as 1 (susceptible). For the 'resistant scale', lines with an average original score of ≤2.5 were scored as 0 (resistant), while the remaining lines were scored as 1 (susceptible). For the 'susceptible scale', lines with an average original score of ≥4 were scored as 1 (susceptible), and the remaining lines as 0 (resistant). For the 'polarized scale', lines with original scores of only 1 or 2 across all replicates by location were scored as 0 (clearly resistant), lines with original scores of 4 or 5 across replicates as 2 (clearly susceptible), and lines with scores that varied between clearly resistant and clearly susceptible across replicates were scored as 1 (ambiguous). Missing data are due to failed germination or lack of growth.

Table S3 SNP markers associated with anthracnose resistance based on a -log_{10}(p-value) above the FDR significance threshold. Data are from the Live Oak, FL and Citra, FL locations, using the polarized scale (Table S2), with heterozygous markers treated as missing. This dataset identifies the SNP marker number (Marker), chromosome on which the SNP marker resides (Chrom), and the position (in basepairs) of the SNP on the chromosome (Genomic Position). The -log_{10}(p-value) for the marker at each location (Live Oak 2013 significance, Citra significance, Live Oak 2015 significance) is denoted with an asterisk to indicate a score above the FDR threshold of 5%. The identity of each GBS-derived marker allele is also provided (Early Hegari-Sart, Bk7).

Table S4 Comparison of the effect on the loci due to the different phenotypic scales and approaches for labeling the heterozygous markers. Each row represents the location of the experiment (Location), the chromosome on which the resistance locus resides (Chrom), the phenotypic scale used for the analysis (Scale; Table S2), and how heterozygous markers were treated (Hets_as): Changed to 'Bk7' alleles (Het=Bk7) or 'Early Hegari-Sart' alleles (Het=EHS), recorded as missing data (Het=miss), or kept as heterozygous (Het=Het). Provided for each association analysis are the FDR thresholds at 5% (FDR), the area of the resistance loci (in Mb) based on a significant association between SNP markers and phenotype (Locus Area), the number of markers within the locus (Number of Markers), the number of markers per Mb (Markers/area), the marker with the lowest p-value given as the $-\log_{10}(p)$ (Highest Score), the identity of the marker with the lowest p-value (Marker Name) and its position (Marker position).

Table S5 Allele-specific PCR to identify 'Bk7'-derived alleles on chromosome 9 in four anthracnose-resistant cultivars. The name (Primer Name) and sequence (Primer Sequence), (listed 5' to 3') for each primer are provided. The GBS-derived SNP markers that formed the basis for the primers are identified by their name (SNP Marker), position on chromosome 9 (Position, in basepair), and the identity of the 'Bk7' allele of the SNP marker (SNP Nucleotide). The presence of a 'Bk7' SNP allele in the cultivars (Cultivars) at the various SNP markers is indicated by a gray "X". The optimal annealing temperature (T_m , °C) for each primer pair and the PCR program are also provided.

Table S6 Complete distribution of phenotypes based on the parental origin of the marker alleles. QTL for each line were classified as either derived from Bk7 (BB) or Early Hegari-Sart (EE), or as missing data (--) due to heterozygosity or ambiguity in the marker data. The results are grouped based on the generation of the population (Population). Because of its size, the QTL on chromosome 7 was separated into two segments, one located on the short arm (Chr7Short), and one on the long arm (Chr7Long). The total number of lines within each genotypic class and the number of lines within each phenotypic class (resistant, ambiguous, susceptible) are provided (Phenotype Count).

Table S7 Proteins and gene ontology groups identified from the sorghum transcripts within the loci of interest. Transcripts were obtained from the annotated sorghum genome v2.4, proteins were identified using BLASTX and gene ontologies were identified using BLAST2GO. The transcript (Seq. Name) and the description of the gene product (Seq. Description) are provided, along with the sequence length (Seq. Length), the number of BLASTX hits (capped at 20; #Hits), the lowest e-value of the BLASTX hits (min. eValue), and the average of the percent similarity scores of the BLASTX hits (mean Similarity). The BLAST2GO results are given with the number of gene ontology results (#GOs) and the list of the gene ontology results (GOs). DNA markers located within the transcripts have been added to facilitate follow-up analysis, with the marker name (Marker), marker chromosome (Chrom), and position of the marker on the chromosome (Position). Annotated genes considered candidate defense-related genes are marked with an asterisk in the last column (Candidate Gene).

Table S8 Curated reference *R*-genes aligned to the identified anthracnose resistance loci. Reference *R*-genes were obtained from Plant Resistance Gene Database and aligned to the sorghum v2.4 reference genome with BLASTN. The results show the Plant Resistance Gene Database identifier (Query), the name of the resistance gene (Gene), the species in which the resistance gene was identified (Species), the sorghum chromosome (Sorghum Chrom; 7 or 9) and the start and end positions (Start Position, End Position) of the putative sorghum *R*-gene ortholog, the e-value (e-value) and the percent similarity (Similarity) generated by BLASTN. If an *R*-gene aligned within a sorghum transcript, the transcript name is provided (Sorghum Transcript).

Table S9 Reference *R*-genes with similarity to the sorghum reference genome identified via BLAST. The chromosome, start position, and end position mark the position in the sorghum genome v2.4 of unannotated candidate *R*-genes, based on similarity to *R*-genes from other species.

Figure S1 Visual representation of the development of the mapping population. The image of the sorghum plant is courtesy of Leana Rosetti and Hesperian Health Guides and is used with permission.

Figure S2 Flowchart for the steps used in the filtering of GBS markers. The different steps in the filtering process are shown on the left, starting at the top. The center and right columns indicate the number of markers left after each filtering step, for the 2013 and 2015 populations, respectively.

Figure S3 Histograms displaying the distribution of plant height across the population in the three environments. Plant height (in m) is the distance from the base of the plant to the top of the panicle. The plant height of the parents falls within in the dark green areas.

Figure S4 Comparison between the marker order on chromosome 7 generated by JoinMap and the marker order based on the reference genome. The GBS-derived SNP markers were first filtered (identical parental alleles, MAF of ≤10%, >80% heterozygosity, an inbred heterozygosity score of <0.8, and markers with identical genomic information) so that 550 markers remained. The deviations from a slope of 1 indicate a mismatch between the two methods or ordering markers. Deviations are due to poor-quality markers, areas of low recombination, the centromere, and the *bmr12* locus. See Figure 1 in the main text for comparison.

Figure S5 Association analysis overlaying different transformations used for association analysis. The X-axis represents the genome location and the Y-axis represents the $-\log_{10}$(p-value) obtained from Fisher's exact test for the 2013 Live Oak experiment. A higher Y-value indicates a stronger association between the marker at that position and disease resistance. The vertical black lines separate each of the 10 sorghum chromosomes, while the colored horizontal lines mark the 5% FDR significance thresholds for the corresponding analysis. **(A)** Association using the 'polarized scale' and the four ways of treating heterozygotes: Classifying a heterozygous marker as homozygous for the 'Bk7' allele (blue) or 'Early Hegari' allele (red), or treating the marker locus in question as missing (green) or as a heterozygote (gold) **(B)** Association between the markers and disease phenotype using the four different resistance scales (see Table S2), with heterozygous markers treated as missing. The blue line represents the 'extremely resistant scale', the red line represents the 'resistant scale', the green line represents the 'susceptible scale', and the gold line represent the 'polarized scale'.

Figure S6 Two-tailed Fisher's exact test for association analysis. The association between SNP markers and the disease phenotype using the 'resistant scale' and heterozygous markers treated as missing for the 2013 Live Oak experiment. A higher Y-value indicates a stronger association between the marker at that position and disease resistance. The vertical black lines separate each of the 10 sorghum chromosomes, while the black horizontal line marks the 5% FDR significance threshold. The blue line represents the left-tailed Fisher's exact test, indicating resistance loci derived from 'Bk7', while the red line represent the right-tailed Fisher's exact test, indicating resistance loci derived from 'Early Hegari'.

File S1

SAS code for the 2013 GBS marker filtering steps and Fisher exact test. The program filters the 130k markers generated at the Institute of Biotechnology at Cornell University into the 5,186 markers used for this study, and performs a Fisher's exact test between each marker and phenotype. The input files needed are Supplemental File S6 (original GBS data), Supplemental File S8 (list of identical markers), and Supplemental Table S2 (transformed phenotypic scales, in tab-delimited text format). The output is a single file with Supplemental File S2 (filtered markers) and Supplemental File S4 ($-\log_{10}(p)$ of Fisher's exact test) combined in one table.

File S2

Final GBS markers from 2013 after all filtering steps are applied. Markers for the F_4 and F_5 mapping populations are provided with the maker name (marker), the sorghum chromosome on which the marker resides (chrom), the marker position on the chromosome (position), and the parental nucleotide for ‘Early Hegari-Sart’ (EH-S) and ‘Bk7’ (Bk7). Genotypic data of the lines are reported with a biparental classification: “a” and “b” indicate alleles derived from ‘Early Hegari-Sart’, and ‘Bk7’ respectively, “h” refers to a heterozygous marker, and “-” indicates markers removed due to their origin from the *bmr12* donor line used to generate EH-S *bmr12*.

File S3

Final GBS markers from 2015 after all filtering steps are applied. Markers for the F_4 mapping population are provided with the maker name (marker), the sorghum chromosome on which the marker resides (chrom), the marker position on the chromosome (position), and the parental nucleic acids for 'Early Hegari-Sart' (EH-S) and 'Bk7' (Bk7). Genotypic data for the individual lines are reported with a biparental classification: "a" means alleles derived from 'Early Hegari-Sart', "b" means 'Bk7' alleles, and "h" means heterozygous calls.

File S4

The –log_{10}(p-value) of the Fisher's exact test for every marker, location in 2013, phenotypic scale, and heterozygous call. Each row represents a different marker (marker with the 32 columns identifying the location as either Live Oak (Live_Oak_...) or Citra (Citra_...). The phenotypic scales are identified as the 'extremely resistant' scale (…_extrmresistant_...), 'resistant' scale (…_resistant_...), 'susceptible' scale (…_susceptible_...), or the 'polarized' scale (…_polarized_...). The method to treat the heterozygous markers are identified as changing the heterozygotes to 'Bk7' alleles (…_H_B), 'Early Hegari-Sart' alleles (…_H_A), missing values (…_H_N), or to leave the heterozygous markers as heterozygotes (…_H_H).

File S5

The –log_{10}(p-value) of the Fisher's exact test for every marker, phenotypic scale, and classification of heterozygous markers for the Live Oak 2015 location. Each row represents a different marker (marker). The phenotypic scales are identified as the 'extremely resistant' scale (…_extrmresistant_...), 'resistant' scale (…_resistant_...), 'susceptible' scale (…_susceptible_...), or the 'polarized' scale (…_polarized_...). The methods to treat the heterozygous markers are identified as changing the heterozygotes to 'Bk7' alleles (…_H_B), 'Early Hegari-Sart' alleles (…_H_A), missing values (…_H_N), or to leave the heterozygous markers as heterozygotes (…_H_H).

File S6

Original GBS data from 2013 in hapmat format. The hapmat format, written as .hmp in the file extension, provides information on the marker name (rs#), the variant nucleotides in the SNP marker (alleles), the chromosome or super contig to which the marker aligned (chrom), the position on the chromosome (pos), whether the sequence aligned to the forward or reverse strand (strand), and the sequencing results for the different lines, including the parents. The column headings for the sequencing data are in a coded format that is changed to the correct line number when imported into the SAS filtering code (Supplemental File S1).

File S7

Original GBS data from 2015 in hapmat format. The hapmat format, written as .hmp in the file extension, provides information on the marker name (rs#), the variant nucleotides in the SNP marker (alleles), the chromosome or super contig to which the marker aligned (chrom), the position on the chromosome (pos), whether the sequence aligned to the forward or reverse strand (strand), and the sequencing results for the different lines, including the parents.

File S8

List of markers with identical GBS genotypic data. This list is for use in the SAS filtering code (Supplemental File S1), and lists markers that contain identical genotypic information as another marker. The marker name (rs#) and the chromosome on which the markers reside (Chrom) are provided.
